# A Rare Case of Atypical Pelvic Retroperitoneal Lipoma Mimicking Liposarcoma; A Case Report and Review of Literature

**DOI:** 10.1002/ccr3.70236

**Published:** 2025-02-20

**Authors:** Bea Harris Forder, Sabina Nistor, Ather Siddiqui, Hooman Soleymani majd

**Affiliations:** ^1^ Medical Sciences Division University of Oxford Oxford UK; ^2^ Department of Gynaecology Oncology Oxford University Hospitals Foundation Trust Oxford UK; ^3^ Nuffield Orthopaedic Centre Oxford University Hospitals Foundation Trust Oxford UK; ^4^ Nuffield Department of Women's and Reproductive Health University of Oxford Oxford UK

**Keywords:** obstetrics/gynecology, oncology, orthopedics, surgery

## Abstract

Retroperitoneal lipomas are exceptionally rare tumors that mimic liposarcomas. We present a case of a woman in her 30s undergoing fertility investigations, with an incidental finding of a retroperitoneal tumor on MRI. PET and MRI scans suggested this may be a liposarcoma, with widespread effects on the surrounding iliac vessels. Collaboration between the orthopedic sarcoma service, gynecological oncology, and vascular surgery was required to excise the tumor without damaging the surrounding vessels. The patient underwent an exploratory midline laparotomy, with mobilization of the ascending colon along the Toldt line to gain access to the tumor. The morbid adherence of the tumor to neurovascular structures added significant complexity to this procedure; however, complete tumor resection was achieved with mobilization and excision of the obturator nerve only. The initial histological diagnosis was well‐differentiated liposarcoma; however, subsequent molecular analysis showed no evidence of MDM2 amplification, suggesting a final diagnosis of benign lipoma. This case exemplifies diagnostic challenges in lipomatous retroperitoneal tumors, as well as the importance of inter‐disciplinary collaboration in surgery to achieve the best surgical and oncological outcomes.

**Summary**

- A surgical multidisciplinary team approach between orthopedic, gynecological oncology and vascular surgeons can enable complete marginal excision of retroperitoneal tumors, improving patient outcomes both around surgery and in terms of long‐term survival.

## 1 Background

Liposarcoma is a tumor derived from mesenchymal fat‐cell precursors, suggested to account for 12.8% of soft tissue tumors [1]. Liposarcomas are typically seen in older patients, aged around 50–65 [2], affecting the soft tissues of the extremities and the retroperitoneum [3], although other regions can be affected. Liposarcoma presents insidiously; it has been shown that 44% of patients with retroperitoneal liposarcoma present asymptomatically, while others complain of pressure effects exerted by the tumor, causing abdominal bloating and pain [4].

Histologically, the World Health Organization (WHO) classifies liposarcoma into four types [5], with drastic differences in prognosis—the 5 year survival rate for a well‐differentiated liposarcoma is 82%, compared to 50% for a de‐differentiated liposarcoma [2]. The management of liposarcoma typically involves surgical resection, with more aggressive subtypes sometimes receiving additional treatment in the form of radiotherapy or chemotherapy [6].

Here, we report a case of an asymptomatic retroperitoneal tumor identified as an incidental finding during investigations for subfertility; this tumor was suspicious for a liposarcoma on imaging. Imaging showed that the tumor was tightly adherent to multiple vascular and nervous structures within the retroperitoneum. Due to the location of the tumor and its relation to surrounding neurovasculature in the pelvis, the sarcoma team sought input from gynecological oncology and vascular surgeons to maximize preservation of these structures, resulting in the best possible outcome for the patient. Initial histology suggested a well‐differentiated liposarcoma, sclerosing variant, but molecular cytogenetic analysis showed a lack of MDM2 amplification, suggesting a final histology diagnosis of benign lipoma.

## 2 Case Presentation

We present the case of a 39‐year‐old woman who was undergoing investigations for fertility problems and was referred to the sarcoma service following an MRI scan showing a retroperitoneal pelvic wall lesion. This lesion was asymptomatic, and the patient had not noticed a mass or any constitutional symptoms. She had a past medical history of obesity, hypothyroidism (for which she took levothyroxine) and iron deficiency anemia, which was managed with iron supplementation. She suffered from fertility problems, having recently trialed letrozole. She was allergic to Penicillin, having had a past reaction of vomiting, and had no surgical history of note. The patient had a body mass index of 40 kg/m^2^, was a current smoker, smoking three cigarettes a day, and has a World Health Organization (WHO) performance status grade of 0, meaning she was able to perform all activities independently and without issues.

## 3 Investigations

**FIGURE 1:**
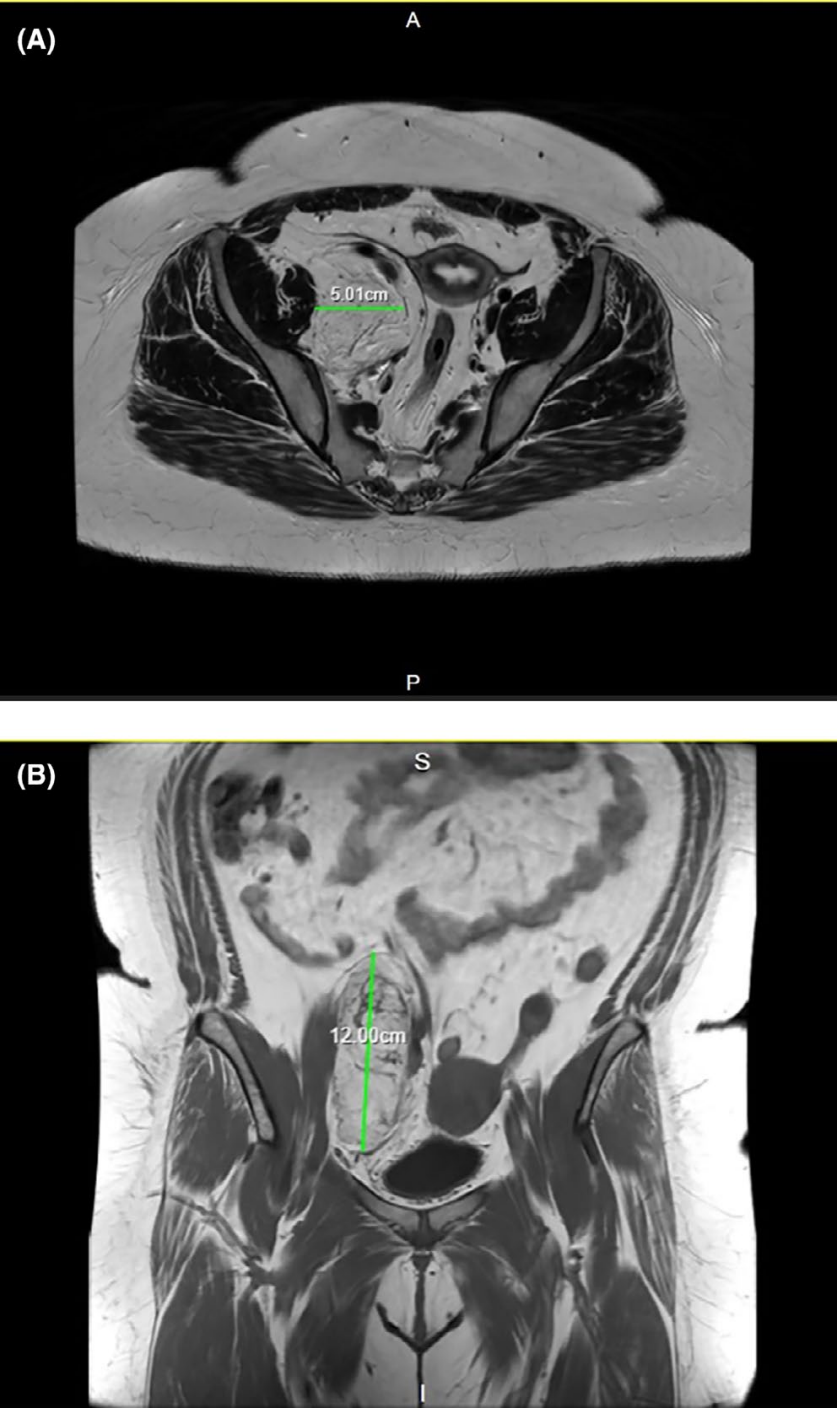
T1 weighted MRI scan demonstrating a large lipomatous mass measuring 120 × 50 × 75 mm medial to the right iliopsoas, displacing the iliac vessels, uterus and bladder without invasion of adjacent muscles and organs. (A) Axial view. (B) Coronal view.

**FIGURE 2:**
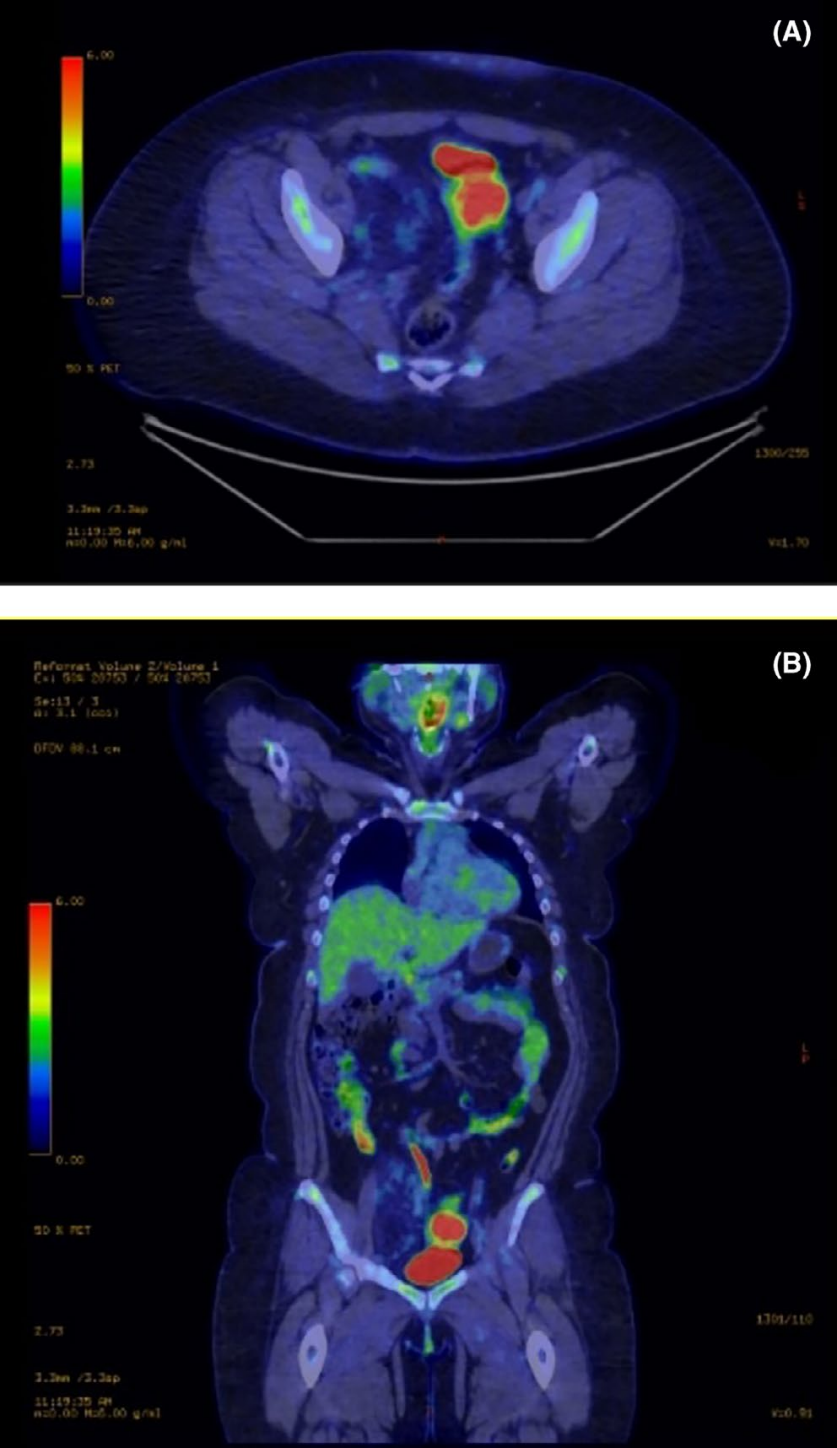
PET CT images showing the right pelvic lipomatous tumor, containing mildly avid soft tissue elements, with no evidence of nodal or metastatic disease. (A) Axial view. (B) Coronal view.

The patient was referred to the sarcoma MDT with an MRI scan (Figure 1) which showed a lipomatous tumor measuring 120 mm in height, 50 mm in width, and 75 mm in depth overlying and medial to the right iliopsoas, displacing the iliac vessels, uterus, and bladder without invasion of adjacent muscles and organs. The tumor was also compressing the common iliac vein at the L5/S1 level. This MRI showed no lymphadenopathy and a normal marrow signal. The MRI was suspicious for a DVT in the right external iliac vein; however, this was later ruled out by a CT venogram. On PET CT (Figure 2), the lipomatous mass contained mildly avid soft tissue elements, and there was no convincing nodal or metastatic disease.

In this case, the radiologists had a high index of suspicion that the diagnosis was liposarcoma based on CT and PET CT findings. A biopsy of the tumor was not recommended, as it was deemed to be high risk for tumor spillage and dissemination. In view of the large tumor size and its location, as well as concerning appearance on imaging, the sarcoma multidisciplinary team (MDT) felt that the mass needed excision regardless of the diagnosis.

## 4 Treatment

The patient was discussed at the sarcoma MDT, which recommended that the patient undergo surgery for marginal excision of the tumor. Due to the location of the tumor within the pelvis and its proximity to the surrounding pelvic and vascular structures, the sarcoma surgeons felt it would be appropriate to perform the surgery in collaboration with gynaeoncology oncology and vascular surgery.

After a general anesthetic, the patient was placed in a modified Lloyd‐Davis position, ensuring the ‘Well‐leg’ checklist [7] was adhered to, and then a midline xiphi‐pubic laparotomy was performed. A right medial visceral rotation was achieved by dividing the peritoneum along the line of Toldt up to the hepatic flexure and medializing the ascending colon (the Cattell—Braasch maneuver). Access to the right pelvic sidewall was gained. The right ureter was identified and found to be traveling along the superior aspect of the tumor; hence, right ureterolysis was performed, and the right ureter was medialized. The inferior vena cava and the aorta were accessed proximally, and the left pelvic and para‐aortic lymph nodes were dissected. While lymph node excision is not normally indicated for liposarcoma in the absence of evidence of nodal involvement, in this case, it was deemed necessary in order to gain better visualization of the pelvic vasculature, with the nodes excised for surgical rather than oncological purposes. The right round ligament was then divided, aiding access to the tumor.

**FIGURE 3:**
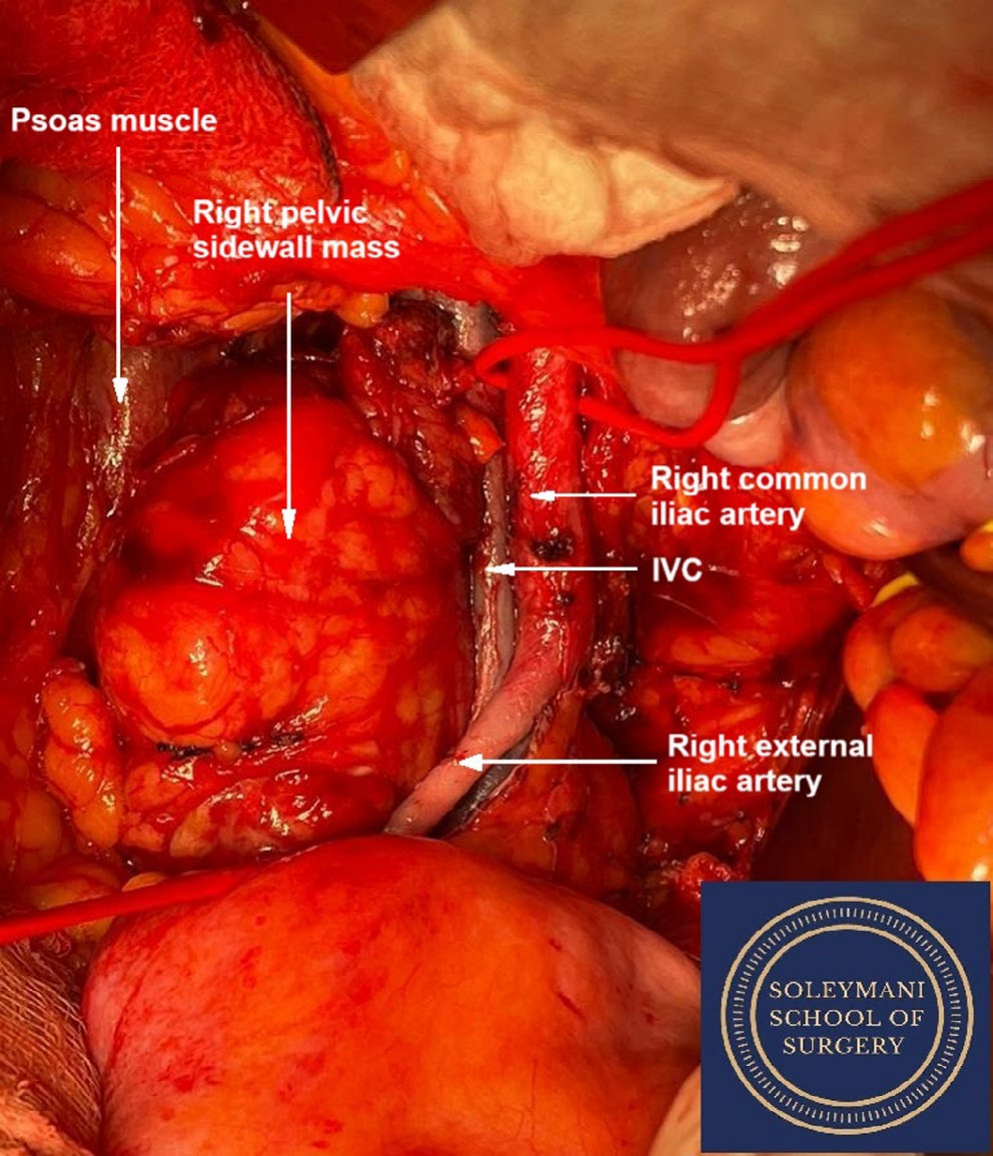
Intraoperative image demonstrating the right pelvic mass lying between the psoas muscle and the right common and external iliac vessels.

The tumor was straddling the vascular structures including the inferior vena cava, the right common and external iliac artery and vein. It was wedged between the right internal iliac artery and vein, and the external iliac artery and vein and inferior vena cava (Figure 3). The tumor was successfully dissected from these vessels through the joint efforts of the four consultant surgeons spanning the different specialties involved. At the point, the lumbo‐sacral trunk and sacral nerve roots were identified.

**FIGURE 4:**
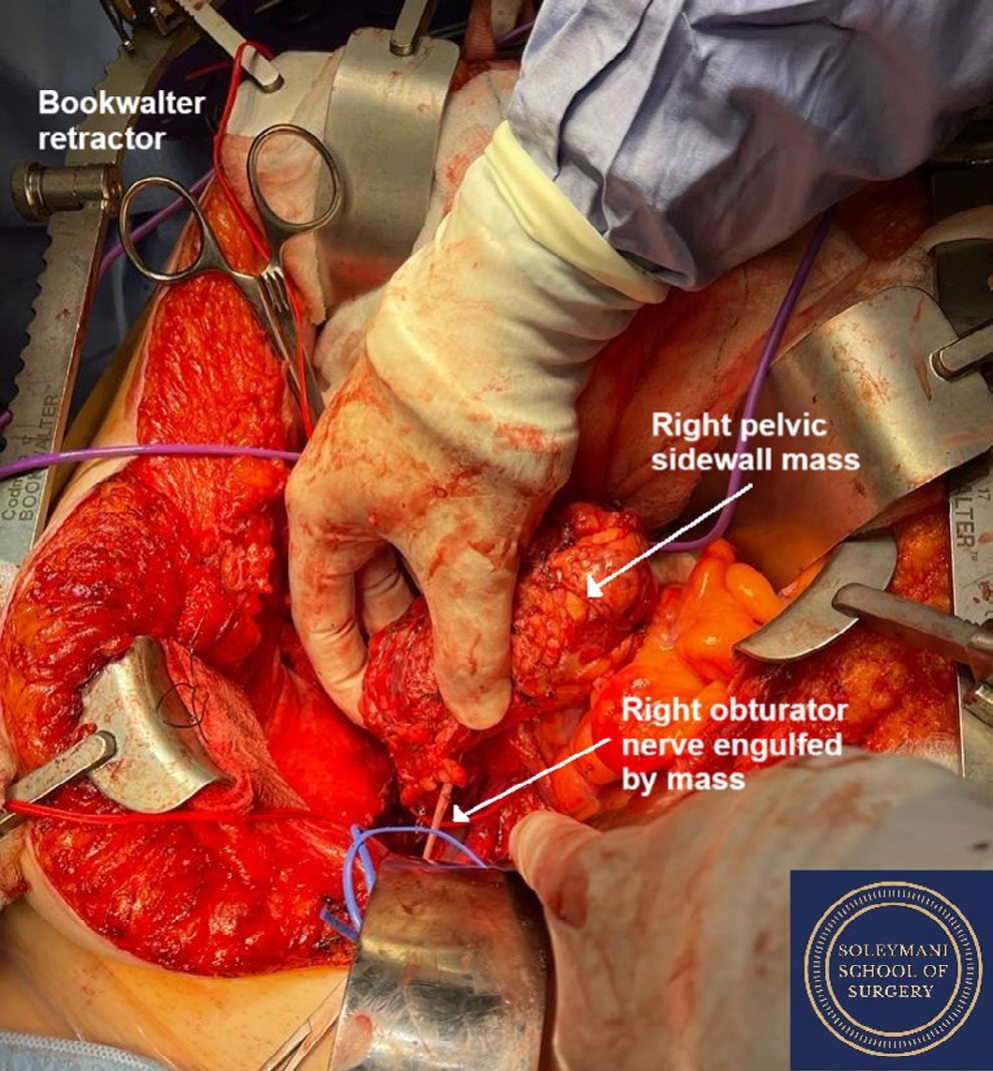
Intraoperative image demonstrating the non‐salvageable obturator nerve engulfed in the tumor.

**FIGURE 5:**
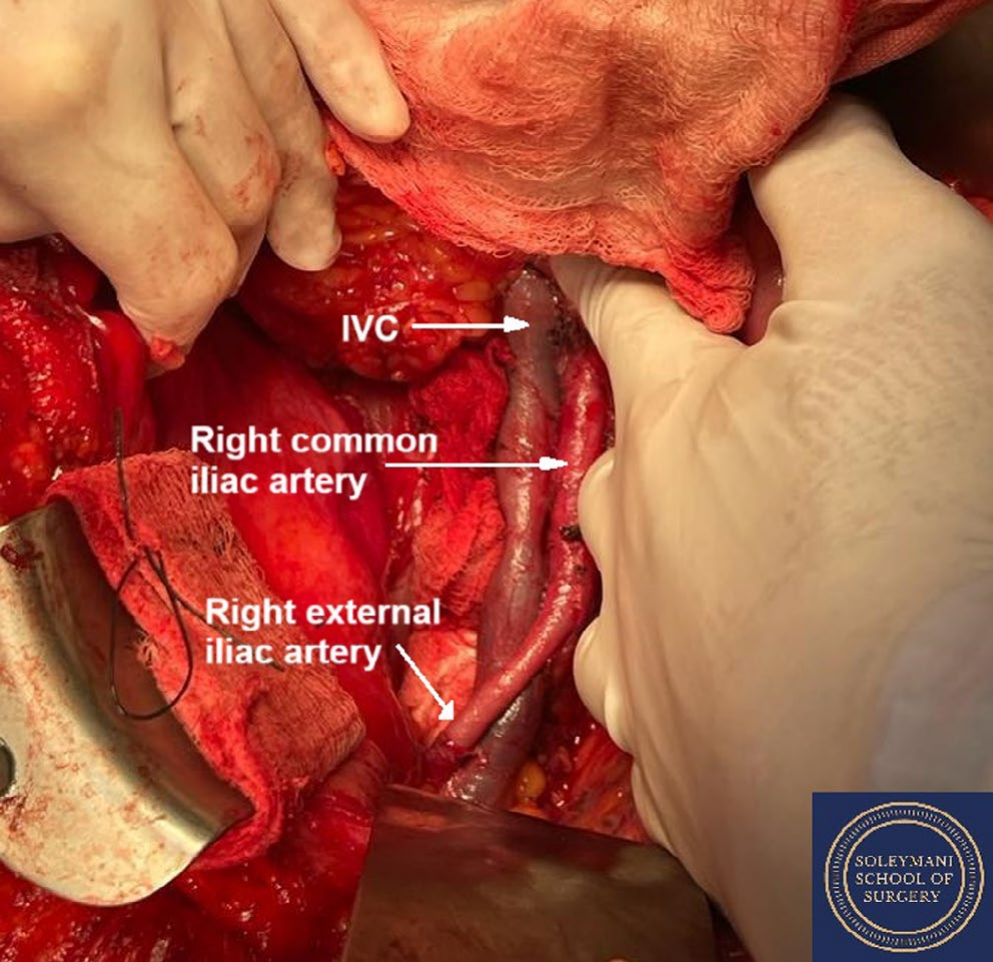
Surgical field following excision of right pelvic mass.

**FIGURE 6:**
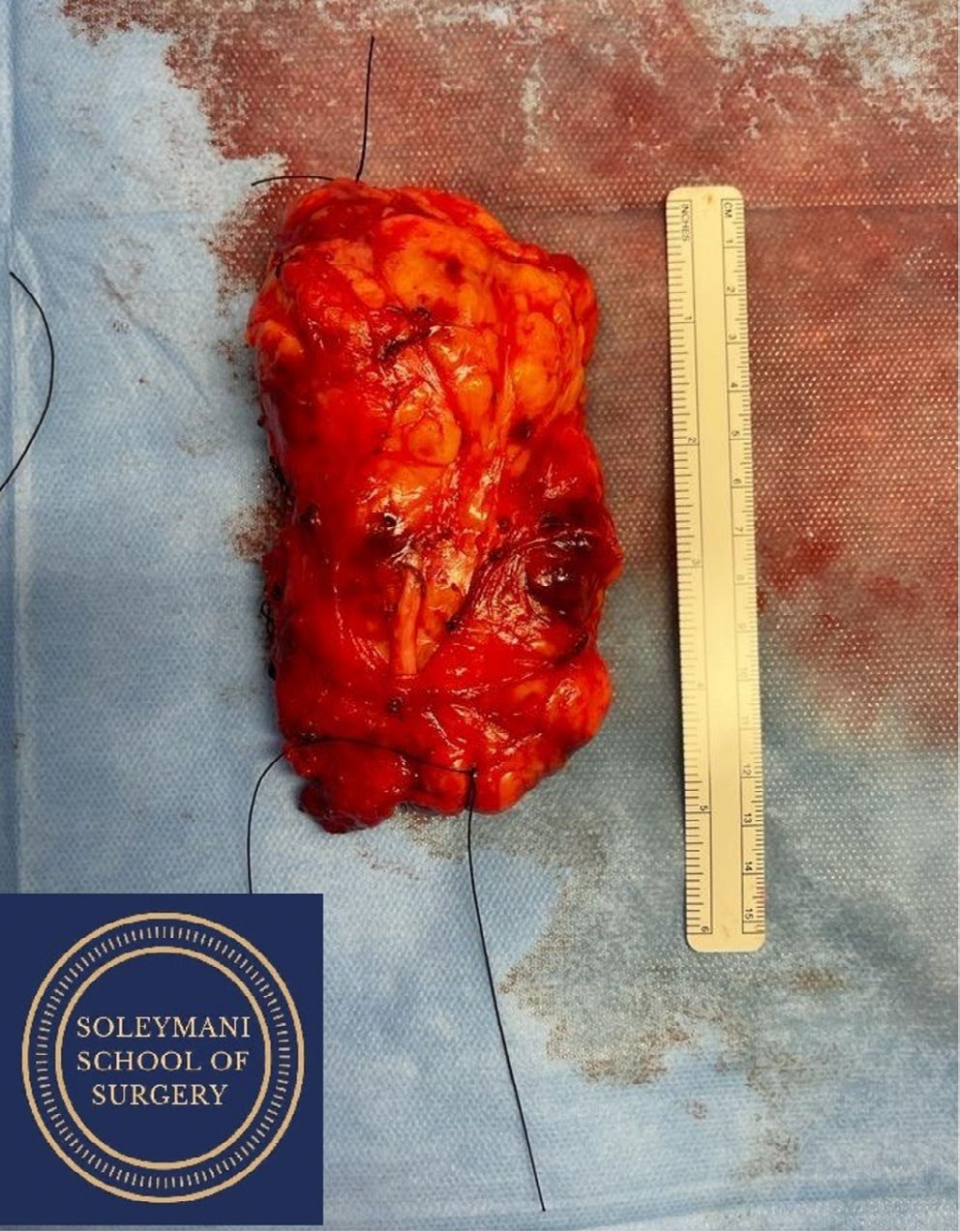
Excised 12 × 5 × 7.5 cm en‐bloc specimen.

The tumor was lying adjacent to numerous muscular structures including iliacus, psoas, quadratus lumborum, and obturator internus. It filled the right lumbo‐sacral space and obturator fossa and was engulfing and densely adherent to the obturator nerve (Figure 4). A joint decision was made to sacrifice the obturator nerve with the tumor, as it was encased and inseparable. The tumor was able to be dissected from the surrounding musculature and bony structures (Figure 5). The specimen was removed en‐bloc with macroscopically clear margins and without spillage (Figure 6).

The right ureter was checked and found to be vermiculating with no evidence of injury or devascularisation. There was no evidence of bleeding from any surrounding vessels at the completion of dissection. A Robinson drain was inserted and left in situ in the pelvic retroperitoneal space.

In total, the operation took 6 h with an estimated blood loss of 550 mL, for which the patient received 230 mL of cell salvage blood. The patient was admitted to a high‐dependency unit postoperatively.

The patient had an uneventful immediate postoperative recovery; however, it was noted 2 days postoperatively that she had some difficulty in right leg adduction secondary to the obturator nerve injury. She also had swelling of the left foot along the 4th and 5th metatarsal joints and 4th and 5th digits. An X‐ray of the left foot and ankle showed no pathology. Upon orthopedic review, it was felt that while these symptoms could be accounted for by lateral ankle ligament sprain, it was important to rule out DVT, although DVT was considered unlikely due to the equalness of calf swelling and no firmness of the calf and was subsequently ruled out by ultrasound doppler.

Other than the presumed left ankle sprain which was managed with an ankle boot and crutches, and a loss of some right adduction, the patient continued to recover well from surgery and was discharged home on the seventh postoperative day with physiotherapy support in place.

## 5 Outcome and Follow‐Up

**FIGURE 7:**
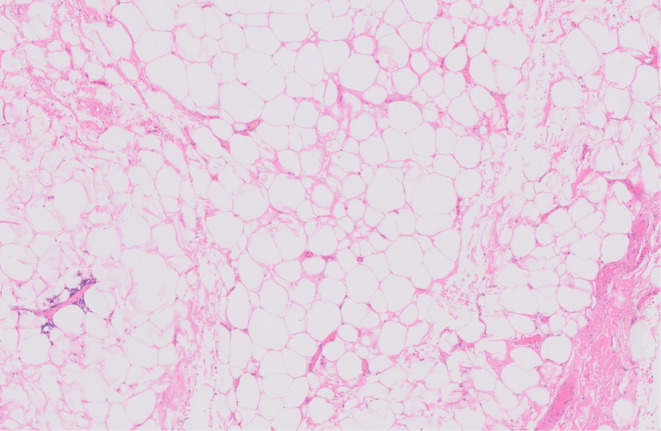
Histopathology: Atypical lipomatous tumor, magnification 40×, Hematoxylin and Eosin (H&E) stain.

Upon review in the gynecological oncology outpatient clinic 2 weeks after discharge, the patient was continuing to recover well, and her loss of right‐sided adduction was improving with physiotherapy. The MDT had discussed histology results from the excised tumor. The initial diagnosis was well‐differentiated liposarcoma, sclerosing variant; however, subsequent molecular cytogenetic investigation showed no evidence of MDM2 amplification in any of the 100 cells sampled, therefore favoring the final diagnosis of a benign lipoma. (Figure 7). The patient will undergo regular 6‐monthly surveillance, including chest X‐rays, under the care of the sarcoma team, with annual MRI.

## 6 Discussion

The great majority of retroperitoneal tumors (80%) are malignant, with liposarcoma representing the most common histological subtype (45%) [8]. The occurrence of retroperitoneal lipomas is extremely rare, with less than 50 cases described in the literature. Lipomas are common encapsulated soft tissue tumors, benign proliferations of mature adipocytes, usually occupying subdermal tissues of the extremities and trunk [9]. Although slow‐growing, they may reach very large dimensions and usually present with abdominal pain or pressure symptoms [10]. Other reported symptoms may be urinary (dysuria, polyuria, urgency, urinary retention and incontinency), gastrointestinal (constipation, tenesmus, diarrhea), venous obstruction and thrombosis, lymphedema, and sciatic pain [9]. Lipomas have no gender predominance and have been described in all age groups, from children to elderly patients [9, 11]. Pelvic lipomas may extend into the inguinal or perineal regions through the sciatic foramen, obturator foramen, or pelvic floor [12].

The differential diagnosis of retroperitoneal lipomas is difficult, and distinction from well‐differentiated liposarcoma poses particular challenges. MRI scans are superior to CT due to better soft tissue contrast resolution. On MRI, lipomas are isointense with fat, with or without a few thin linear internal septations [8]. Features favoring liposarcoma on CT and MRI are the following: size > 10 cm, presence of thick septa, presence of globular and/or nodular nonadipose areas, and a lesion containing less than 75% fat [13]. On PET CT, lipomas and atypical lipomatous tumors typically demonstrate a much lower maximum standardized uptake value (SUV_*max*_) than liposarcomas [14].

Percutaneous biopsy is a controversial approach due to the risk of local spread by the implantation of malignant cells along the puncture route should it be a case of liposarcoma. We opted not to obtain a biopsy, and surgical resection was achieved with clear margins and an intact capsule.

On histological examination, well‐differentiated liposarcoma has characteristic irregular, finely fibrillar fibrous septa containing enlarged hyperchromatic cells and rare lipoblasts, features which are absent in lipomas. However, some lipoma‐like well‐differentiated liposarcomas have extremely few diagnostic hyperchromatic cells and lipoblasts and are hence extremely difficult to distinguish confidently from lipoma [15].

In such cases, molecular cytogenetic analysis, including fluorescence in situ hybridisation (FISH) for MDM2 amplification, is essential. Cytogenetically, lipomas usually show rearrangements of chromosome 12q15 with a variety of chromosome partners, whereas well‐differentiated liposarcoma shows amplification of this and surrounding areas, often in the form of giant marker and ring chromosomes. The amplified region contains several genes, including MDM2, carboxypeptidase M (CPM), sarcoma aplified sequence (SAS), cyclin‐dependent kinase 4 (CDK4), DNA damage‐inducible transcript 3 (DDIT3/CHOP) and high‐mobility group AT‐hook 2 (HMGA2)(15).

In our case, MRI, PET CT, and histopathology could not differentiate clearly between lipoma and well‐differentiated liposarcoma, and the final diagnosis of retroperitoneal lipoma could be made only on cytogenetic analysis, which failed to identify any MDM2 amplification.

A particularly challenging element of this case was the tumor adherence to major neurovascular structures of the pelvis, in particular the inferior vena cava, the right common iliac and right external iliac arteries and veins, the right lumbo‐sacral trunk, and the right obturator nerve. Dissection with optimal margins was therefore a complex task requiring input from multiple surgical specialties to minimise collateral damage and ensure a safe approach by preserving vital pelvic structures. In this case, all structures except the obturator nerve were preserved, allowing minimal loss of function to the patient whilst maintaining oncological clearance to optimise patient outcome. Pathology of the retroperitoneum is commonly dealt with by the gynaecological oncology team, due to our regular exposure to pelvic sidewall surgery, We have previously described our team's involvement in the surgical management of two rare cases of retroperitoneal leiomyomatosis [16, 17].

Whilst from an organisational point of view, a surgical approach spanning multiple specialties can evoke logistical problems such as those related to scheduling, with regard to surgical and patient outcomes this approach is considered vital [18]. The effect of a multidisciplinary approach to surgical cancer care is measurable; it has been shown to decrease perioperative [19] and postoperative mortality [20] and improve long‐term survival [19].

## 7 Conclusion

In summary, we present the case of a large retroperitoneal lipoma and discuss the diagnostic challenges posed by this exceptionally rare tumor. The excision of this mass, closely adherent to key neurovascular pelvic structures, exemplifies a successful multidisciplinary surgical effort. By collaboration between different surgical specialties, the entire tumor was removed without opening the capsule, with conservation of all essential structures except for the right obturator nerve, and the patient will be minimally impacted with ongoing physiotherapy.

## Author Contributions

**Bea Harris Forder:** formal analysis, project administration, visualization, writing – original draft, writing – review and editing. **Sabina Nistor:** conceptualization, data curation, investigation, methodology, project administration, writing – original draft, writing – review and editing. **Ather Siddiqui:** conceptualization, data curation, investigation, methodology, writing – review and editing. **Hooman Soleymani majd:** conceptualization, investigation, methodology, formal analysis, project administration, writing – review and editing.

## Ethics Statement

The authors have nothing to report.

## Consent

Written informed patient consent was gained for the submission of this case report.

## Conflicts of Interest

The authors declare no conflicts of interest.

## Data Availability

Data sharing is not applicable to this article as no new data were created or analyzed in this study.
